# Fluorescent Nanoparticle Uptake by Myzocytosis and Endocytosis in *Colpodella* sp. ATCC 50594

**DOI:** 10.3390/microorganisms11081945

**Published:** 2023-07-29

**Authors:** Tobili Y. Sam-Yellowe, Mary M. Asraf, John W. Peterson, Hisashi Fujioka

**Affiliations:** 1 Department of Biological, Geological and Environmental Sciences, Cleveland State University, Cleveland, OH 44115, USA; m.m.asraf@vikes.csuohio.edu; 2 Cleveland Clinic Lerner Research Institute, Cleveland, OH 44195, USA; peters@ccf.org; 3 Cryo-EM Core, Case Western Reserve University, Cleveland, OH 44106, USA; hxf3@case.edu

**Keywords:** Apicomplexa, *Colpodella* species, *Colpodella* sp. (ATCC 50594), endocytosis, food vacuole, life cycle, myzocytosis, fluorescent nanoparticles, trichrome stain

## Abstract

*Colpodella* sp. (ATCC 50594) is a free-living biflagellate predator closely related to pathogenic Apicomplexa such as *Plasmodium*, *Cryptosporidium* and *Toxoplasma gondii*. *Colpodella* sp. (ATCC 50594) obtain nutrients by preying on *Parabodo caudatus* using myzocytosis. The organization of the myzocytic apparatus and the mechanism of nutrient uptake into the posterior food vacuole of *Colpodella* species is unknown. In this study, we investigated myzocytosis using light and transmission electron microscopy. We investigated the uptake of 40 nm and 100 nm fluorescent nanoparticles and *E. coli* BioParticles by *Colpodella* sp. (ATCC 50594) in a diprotist culture. Transmission electron microscopy was used to investigate the morphology of the tubular tether formed during myzocytosis. *E. coli* BioParticles were taken up by *P. caudatus* but not by *Colpodella* sp. (ATCC 50594). Both protists took up the 100 nm and 40 nm beads, which were observed distributed in the cytoplasm of free unattached *Colpodella* sp. (ATCC 50594) trophozoites, and also in feeding *Colpodella* sp. (ATCC 50594) trophozoites and in the pre-cysts. Fragments of the nucleus and kinetoplast of *P. caudatus* and the nanoparticles were identified in the tubular tether being aspirated into the posterior food vacuole of *Colpodella* sp. (ATCC 50594). Unattached *Colpodella* sp. (ATCC 50594) endocytose nutrients from the culture medium independently from myzocytosis. The mechanisms of myzocytosis and endocytosis among *Colpodella* species may provide important insights into nutrient uptake among the pathogenic apicomplexans.

## 1. Introduction

*Colpodella* species are free-living alveolate predators closely related phylogenetically to the pathogenic Apicomplexa [1,2]. Among the alveolates, *Colpodella* species, along with *Voromonas pontica* and the dinoflagellates *Perkinsus* and *Psammosa* species, possess two heterodynamic flagella and apical complex organelles, and are known as myzozoans [3]. The apical complex organelles, including the rhoptries, micronemes, polar rings and conoid participate in host cell invasion and nutrient uptake among the apicomplexans [4,5,6]. The apicomplexans and apicomplexan-like organisms are a polyphyletic group, thought to have evolved independently, at least three times [2]. *Colpodella* species possess an open conoid, known as a pseudoconoid [3]. Contents of the rhoptries are also used for nutrient acquisition in *Plasmodium* species and among the gregarines [7,8]. *Colpodella* species feed using a process known as myzocytosis, a characteristic of the myzozoans, where they attach to their flagellate and algae prey and aspirate the cytoplasmic contents into a posterior food vacuole. At the conclusion of feeding, most *Colpodella* species encyst. Variations exist in different *Colpodella* species regarding cyst formation. Resting cysts are found in the life cycle of *Colpodella pseudoedax*, in addition to division by longitudinal fission, *C. unguis* divides by oblique-transversal fission and *C. edax* does not encyst [9,10]. Ectoparasitic species such as *C. tetrahymenae* and *C. gonderi* prey on ciliates [3,11] and remain attached to their prey for prolonged feeding [11], after which, encystation occurs in some species [3]. It is unknown whether *Colpodella* trophozoites acquire nutrients using endocytosis in addition to myzocytosis. Roles for endocytosis in nutrient acquisition and membrane homeostasis have been suggested. However, the presence of the inner membrane complex (IMC) found in all apicomplexans may hinder membrane invagination and endocytosis [12]. The micropore found in all apicomplexans is considered a likely structure for endocytosis and in *T. gondii*, the micropore has been reported to be involved in endocytosis and nutrient acquisition [13]. Two human *Colpodella* infections have been described with red blood cells infected [14] and in a tick-borne infection [15]. The mode of infection, stages causing pathogenesis, stages responsible for transmission and nutrient acquisition within the infected host are unknown. Different modes of feeding are known among the Apicomplexa and involve the use of the cytostome in *Plasmodium*-infected red blood cells [12], a mucronal vacuole among archigregarines and blastogregarines [16,17] and micropores, which are found in all Apicomplexans. Endocytosis among the Apicomplexa is not well understood. However, recent reports indicate that endocytosis may be required for membrane homeostasis and recycling in *Toxoplasma gondii* and endocytosis at the cytostome in *P. falciparum* may contribute to artemisinin resistance [12]. The uptake of extracellular material by eukaryotic cells that includes phagocytosis or pinocytosis is categorized under endocytosis and can include receptor-mediated uptake of extracellular material [12]. The types of endocytic processes described in mammalian cells have not been described among free-living protists. A mode of feeding based on “membrane nibbling” known as trogocytosis has been reported in *P. falciparum* and *Toxoplasma gondii* [18]. However, the mechanism is poorly understood. *Selenidium pendula* Giard, 1884 feeds by myzocytosis; however, unlike *Colpodella* sp. (ATCC 50594), the food vacuoles along with rhoptries are located in the anterior end of the trophozoite [17]. The mechanisms of nutrient uptake by endocytosis and myzocytosis are unknown among the colpodellids. Although myzocytosis is thought to be the link between ancestral feeding mechanisms in colpodellids, gregarines and the pathogenic apicomplexans [19], very little information is available regarding the mechanisms of myzocytosis. In this study, we investigated nutrient uptake by *Colpodella* sp. (ATCC 50594) trophozoites using carboxylated-modified fluorescent polystyrene nanoparticles of 40 nm and 100 nm and fluorescent *E. coli* BioParticles. We performed transmission electron microscopy to investigate the ultrastructure of the initial myzocytic contact between predator and prey and the tubular tether formation. The size of nutrients aspirated during myzocytosis has not been studied previously in *Colpodella* species.

## 2. Materials and Methods

**Diprotist culture conditions.** *Colpodella* sp. (ATCC 50594) with *Parabodo caudatus* as prey was obtained from the American type culture collection (ATCC) (Manassas, VA, USA). Predator and prey were cultured together at 24 °C in tissue culture flasks containing Hay medium (Wards Scientific Rochester, New York, NY, USA) bacterized with *Enterobacter aerogenes* as described [20,21]. Cultures were observed and examined using an inverted microscope.

**Staining and light microscopy**. Cells in tissue culture flasks were fixed directly by adding an equal volume of 10% formalin for a final concentration of 5% in the flask (cells “fixed in flight”) as described previously [20,21]. Formalin-fixed cells were stained with Sam-Yellowe’s trichrome stains [22] followed by observations using light microscopy. Fast green (0.5%) in alcohol) was used in place of brilliant green in some staining protocols. All stained smears were examined under oil immersion at ×1000 magnification and images were captured using an Olympus BX43 compound microscope attached to an Infinity HD Lumenera digital camera and Olympus U-TV0.35xc-2 adapter using Infinity HD Capture software.

**Fluorescent Nanoparticle Uptake.** *Colpodella* sp. (ATCC 50594) was cultured at 24 °C with the prey protist *Parabodo caudatus* in *Enterobacter aerogenes* bacterized Hay medium. At 27–28 h post subculture, FluoSpheres and *E. coli* BioParticles (ThermoScientific™, Waltham, MA, USA) were added to the cultures and used to evaluate nutrient uptake during myzocytosis, as well as the size of nutrients taken up by predator and prey. To avoid aggregation, FluoSpheres were initially diluted (1:10) and sonicated in a water bath. Dilutions less than 1:10 resulted in a high concentration of beads contributing to a high background and dilutions higher that 1:10 resulted in a dilute bead concentration in culture. For two 20 mL cultures, 20 µL of FluoSpheres and 180 µL of hay medium was added to microcentrifuge tubes and sonicated in a water bath for 10 min. After subculture, FluoSpheres of 4 µm fluorescent red sulfate microspheres (Ex/Em: 580/605 nm), 2 µm yellow-green fluorescent sulfate microspheres (Ex/Em: 505/515 nm), 100 nm red fluorescent Carboxylate-Modified Microspheres (Ex/Em: 580/605 nm), 40 nm, yellow-green fluorescent Carboxylate-Modified Microspheres (Ex/Em: 505/515 nm) were further diluted 1:1000 in Hay medium followed by sonication to disperse the particles. Concentrations of 50 or 100 µL beads from the initial dilution were added to 10 mL of culture and incubated for 1 h at 24 °C to determine the optimum dilution for uptake experiments. A concentration of 50 µL resulted in a dilute bead suspension so 100 µL was used for uptake experiments. pHrodo Red *E. coli* BioParticles (1 mg/mL) (Ex/Em: 560/585 nm) were diluted by adding 2 mL of hay medium to each 2 mg vial (following manufacturer’s protocols) and sonicated in a water bath for 10 min. An amount of 500 µL of cells were added to 10 mL cultures and incubated for 1h. Cultures were fixed with 5% formalin for 10 min at RT, then centrifuged at 3500 rpms for 10 min in a refrigerated centrifuge. After discarding the supernatant, the pellet was washed by resuspension in 1X PBS, followed by centrifugation. The wash supernatant was discarded and the pellet was resuspended in 1X PBS, for preparation of smears on glass slides. Smears were air-dried and mounted in mounting medium by adding 100 µL of Fluoromount-G containing DAPI (4′, 6-diamidino-2-phenylindole). Smears of formalin-fixed cells were also incubated with CellMask Green actin tracking stain (ThermoFisher Scientific) for cells incubated with 100 nm fluorescent nanoparticles and *E.coli* BioParticles, and CellMask Deep Red actin tracking stain (ThermoFisher Scientific) for cells incubated with 40 nm nanoparticles. Smears were mounted in mounting medium by adding 100 µL of Fluoromount-G containing DAPI (4′, 6-diamidino-2-phenylindole). Slides were sealed with two coats of clear nail polish and slides were examined by confocal and differential interference contrast microscopy. Slides were examined at the imaging core (Lerner Research Institute, Cleveland Clinic, Cleveland, OH, USA). Confocal, fluorescent and differential interference contrast (DIC) images were collected using a Leica SP8 True Scanning Confocal (TCS) DM18inverted microscope (Leica Microsystems, GmbH, Wetzlar, Germany). Stained and confocal images were adjusted to 300 dpi using the CYMK color mode, RGB color mode, auto color and auto contrast on Adobe Photoshop (CC). 

**Electron microscopy.** An aliquot of *Colpodella* sp. (ATCC 50594) in culture medium was added to an equal volume of 8% paraformaldehyde in 0.1 M cacodylate buffer and spun down for 10 min at 3500 rpm. The cell pellet was fixed with 2.5% glutarladehyde, 2% paraformaldehyde in 0.1 M cacodylate buffer. The fixation continued in the same fixative solution for a total of 2 h at room temperature. The pellets were thoroughly rinsed in 0.1 M cacodylate buffer, and then postfixed for 2 h in an unbuffered 1:1 mixture of 2% osmium tetroxide and 3% potassium ferrocyanide. After rinsing with distilled water, the specimens were soaked overnight in an acidified solution of 0.25% uranyl acetate. After another rinse in distilled water, they were dehydrated in ascending concentrations of ethanol, passed through propylene oxide, and embedded in a EMbed 812 embedding media (Electron Microscopy Sciences, Hatfield, PA, USA). Thin sections (70 nm) were cut on a RMC MT6000-XL ultramicrotome. These were mounted on T-300 mesh nickel grids (Electron Microscopy Sciences, PA) and then sequentially stained with acidified methanolic uranyl acetate and stable lead staining solution. These were coated on a Denton DV-401 carbon coater (Denton Vacuum LLC, NJ), and observed in a FEI Tecnai Spirit (T12) transmission electron microscope with a Gatan US4000 4kx4k CCD.

## 3. Results

**Figure 1:**
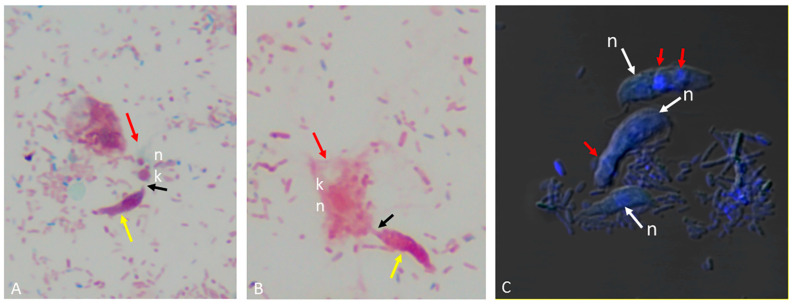
(**A**) Sam-Yellowe’s trichrome A staining of formalin-fixed *Colpodella* sp. (ATCC 50594) (yellow arrow) in myzocytosis with a juvenile *P. caudatus* trophozoite (red arrow). The black arrow shows the tubular tether between predator and prey. (**B**) Sam-Yellowe’s trichrome J staining of formalin-fixed *Colpodella* sp. (ATCC 50594) trophozoite (yellow arrow) in myzocytosis with a mature *P. caudatus* trophozoite (red arrow). (**C**) Confocal microscopy and differential interference contrast (DIC) image of young unattached *Colpodella* sp (ATCC 50594) trophozoites. DAPI-stained material (red arrows) was observed in the cytoplasm in an area below the nucleus (N, white arrows), kinetoplast (K).

Myzocytosis and tubular tethers are shown in predator–prey attachments with Sam-Yellowe’s trichrome staining in Figure 1A,B.

**Figure 2:**
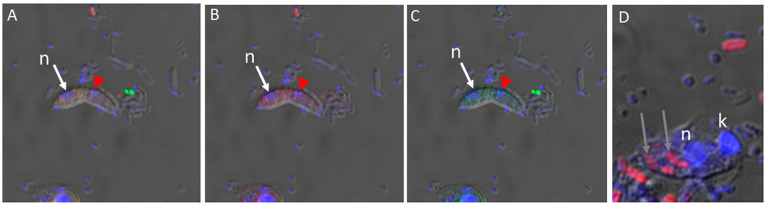
Confocal and DIC images of young fusiform *Colpodella* sp. (ATCC 50594) trophozoites (**A**–**C**) following uptake of 100 nm nanoparticles with CellMask actin green and P. caudatus (**D**) uptake of *E. coli* BioParticles. (**A**) Overlay of DIC, 100 nm nanoparticles, CellMask actin green. (**B**) Overlay of DIC, DAPI, and 100 nm nanoparticles. (**C**) Overlay of DIC, DAPI, and CellMask actin green. (**D**) Overlay of DIC, DAPI and *E. coli* BioParticles (red, gray arrows) in cytoplasm of *P. caudatus*. Red arrowheads label regular bacteria in *Colpodella* sp. (ATCC 50594). White arrows indicate *Colpodella* sp. (ATCC 50594) nucleus (N), kinetoplast (K).

**Figure 3:**
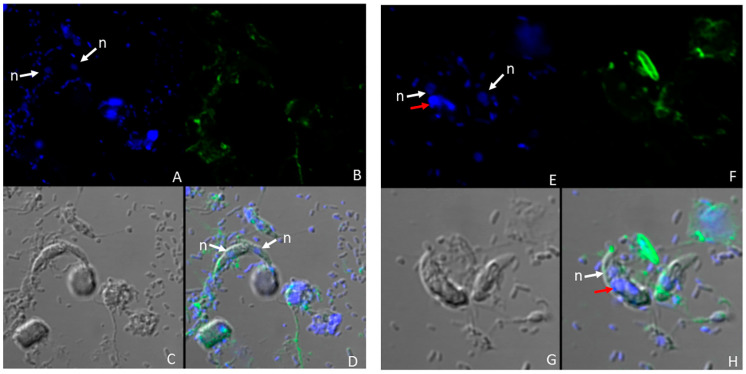
Confocal microscopy and DIC of young *Colpodella* sp. (ATCC 50594) trophozoites following uptake of 40 nm nanoparticles. DAPI-stained nuclei of *Colpodella* sp. (ATCC 50594) trophozoites (white arrows) (**A**,**E**) and 40 nm nanoparticles in the cytoplasm of *Colpodella* sp. (ATCC 50594) trophozoites (**B**,**F**). DIC images of young *Colpodella* sp. (ATCC 50594) trophozoites (**C**,**G**), overlay of DIC, DAPI and 40 nm nanoparticles in unattached *Colpodella* sp. (ATCC 50594) trophozoites (**D**,**H**). DAPI-stained nucleus and kinetoplast of *P. caudatus* taken up into the cytoplasm of *Colpodella* sp. (ATCC 50594) is indicated by the red arrows. The nucleus (n) of *Colpodella* sp. (ATCC 50594) is shown above the nucleus and kinetoplast of *P. caudatus* taken up by *Colpodella* sp. (ATCC 50594) (**H**).

Different sizes of the prey targeted by *Colpodella* sp. (ATCC 50594) trophozoites are shown in both panels. We reported in previous studies the presence of DAPI-stained contents in the food vacuole of *Colpodella* sp. (ATCC 50594) trophozoites [23]. In active cultures, free unattached *Colpodella* sp. (ATCC 50594) trophozoites were identified with DAPI-stained contents in the cytoplasm as shown in Figure 1C. *Colpodella* sp. (ATCC 50594) trophozoites in active culture were observed to attach to and detach from prey after making contact unlike the irreversible attachments of zoites during host cell invasion in pathogenic Apicomplexans such as *Plasmodium* species. We performed nutrient uptake experiments using fluorescent particles of 4 µm, 2 µm, 100 nm and 40 nm and *E. coli* BioParticles in order to determine the size of nutrients taken up in culture by *Colpodella* sp. (ATCC 50594) and its prey. The 4 and 2 µm particles were not taken up by *Colpodella* sp. (ATCC 50594). Fluorescent nanoparticles of 100 nm (Figure 2A–C) and 40 nm (Figure 3A–H) were observed in the cytoplasm of *Colpodella* sp. (ATCC 50594) trophozoites, distributed within the cytoplasm. *Colpodella* sp. (ATCC 50594) trophozoites did not take up *E. coli* BioParticles. *Parabodo caudatus* trophozoites did not take up 4 µm or 2 µm particles but took up 100 nm, 40 nm and *E. coli* BioParticles from the culture. *E. coli* BioParticles were observed in the cytoplasm of *P. caudatus* (Figure 2D, grey arrows).

**Figure 4:**
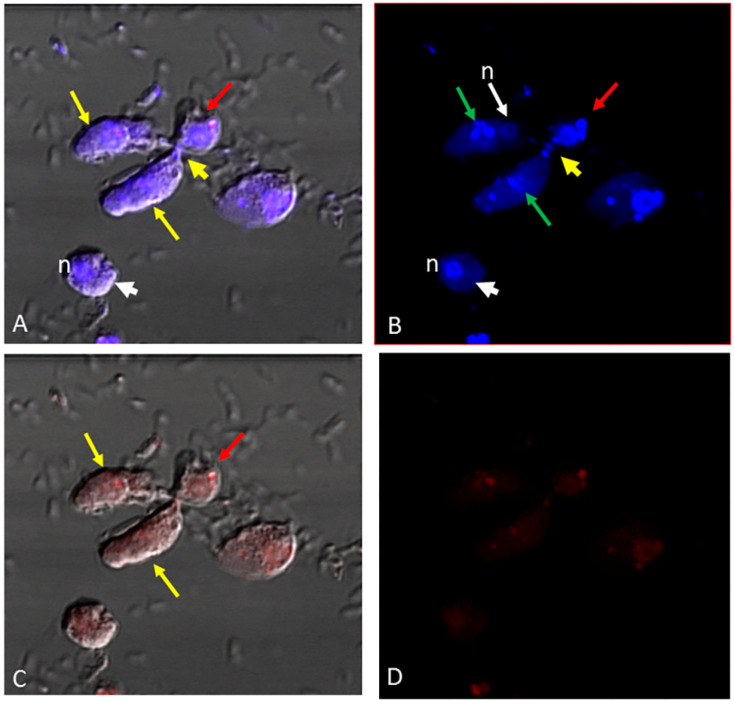
Confocal microscopy and DIC images of two *Colpodella* sp. (ATCC 50594) trophozoites (yellow arrows) and *P. caudatus* trophozoite (red arrow) in myzocytosis. 100 nm nanoparticles (red) are shown distributed in the cytoplasm of predator and prey. (**A**) Overlay of DIC, DAPI and 100 nm beads showing the tubular tether (yellow arrowhead) containing DAPI-stained material. White arrow head shows a single nucleus. *Colpodella* sp. (ATCC 50594) cyst. (**B**) DAPI-stained nuclei and kinetoplast of predator and prey. The DAPI-stained contents aspirated into the food vacuole of *Colpodella* sp. (ATCC 50594) trophozoites is indicated by the green arrows. DAPI-stained nuclear and kinetoplast aspirates are shown in the tubular tether (yellow arrowhead) in transit to the cytoplasm of *Colpodella* sp. (ATCC 50594). (**C**) Overlay of DIC and 100 nm beads showing distribution of beads in the cytoplasm of predator and prey. (**D**) Distribution of 100 nm beads in the cytoplasm of predator and prey.

**Figure 5:**
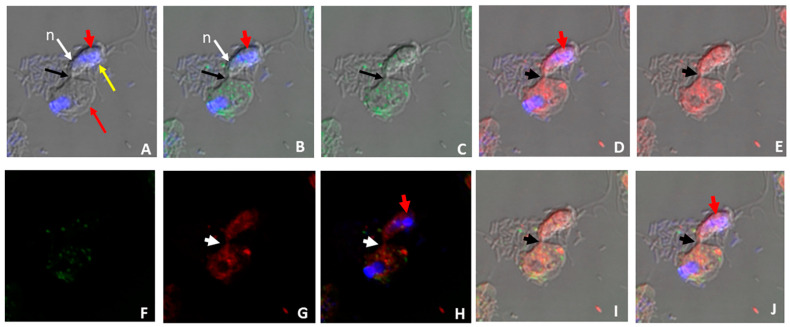
Confocal microscopy, and DIC images of *Colpodella* sp. (ATCC 50594) (yellow arrow) and *P. caudatus* trophozoites (red arrow) in myzocytosis. Following uptake of 40 nm nanoparticles and formalin fixation, cells were stained with CellMask actin red. (**A**) Overlay of DIC- and DAPI-stained cells showing *Colpodella* sp. (ATCC 50594) trophozoite nucleus (white arrow) and DAPI-stained nucleus and kinetoplast of the prey in the posterior food vacuole of *Colpodella* sp. (ATCC 50594) (red arrowhead). (**B**) Overlay of DIC, DAPI and 40 nm beads (green). (**C**) Overlay of DIC and 40 nm beads (green). (**D**) Overlay of DIC, CellMask actin red and DAPI. (**E**) Overlay of DIC and CellMask actin red. (**F**) Distribution of 40 nm beads in the cytoplasm of predator and prey. (**G**) Distribution of CellMAsk actin red in the cytoskeleton of predator and prey and in the tubular tether between predator and prey. (**H**) Overlay of DAPI, CellMask and 40 nm beads. (**I**) Overlay of DIC, CellMask actin red and 40 nm beads. (**J**) Overlay of DIC, DAPI, CellMask actin red, and 40 nm beads. The red arrowheads show the nucleus and kinetoplast of *P. caudatus* in the posterior food vacuole of *Colpodella* sp. (ATCC 50594) (**A**,**B**,**D**,**H**,**J**). The distribution of 40 nm beads (green) in the cytoplasm of predator and prey is shown (**B**,**C**,**F**). CellMask actin red distribution in the cytoskeleton and around the tubular tether (black arrowhead) is shown (**D**,**E**,**G**,**I**,**J**). Overlay of DIC, DAPI, 40 nm beads and CellMask actin (red) (**J**).

The nucleus and kinetoplast of *P. caudatus* were observed in the cytoplasm of an unattached *Colpodella* sp. (ATCC 50594) trophozoite below the nucleus (n) (Figure 3H). In Figure 4, aspirated DAPI-stained nutrients were identified in the cytoplasm and food vacuole of two trophozoites (yellow arrows) attached to *P. caudatus* (red arrow), and in the tubular tether, along with 100 nm nanoparticles (red) also distributed in the cytoplasm of predator and prey (Figure 4B, green arrows show DAPI-stained nutrients and yellow arrow head indicates aspirated nutrients in the tubular tether). Following uptake of nanoparticles and fixation of cells, fluorescent CellMask actin was applied to the smear and the distribution of actin relative to the distribution of nanoparticles was observed. In differential interference contrast (DIC) images showing DAPI staining (Figure 5A), DAPI-stained nucleus and kinetoplast from the prey was identified in the forming posterior food vacuole. Forty nanometer particles (green) as well as DAPI-stained nucleus and kinetoplast was observed in the cytoplasm of *Colpodella* sp. (ATCC 50594) trophozoites and in the forming posterior food vacuole (Figure 5B,C,F). CellMask red actin label was observed in the cytoskeleton of predator and prey and in the tubular tether (Figure 5D,E,G–J).

**Figure 6:**
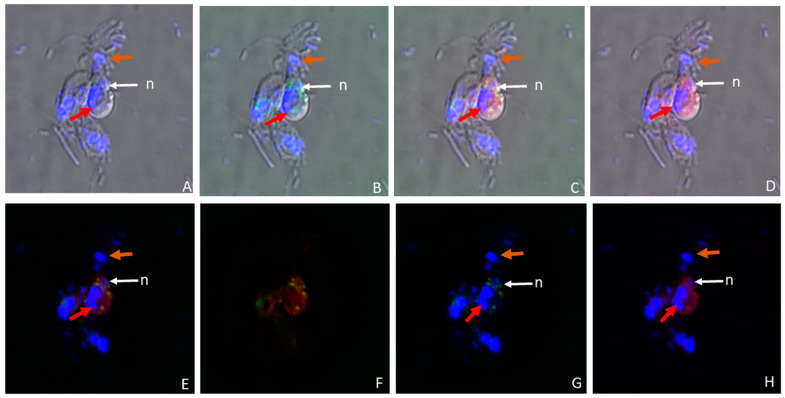
Confocal microscopy and DIC images of *Colpodella* sp. (ATCC 50594) pre-cyst with posterior food vacuole containing 40 nm beads (green) and the nucleus and kinetoplast of *P. caudatus* (red arrow). The nucleus of *Colpodella* sp. (ATCC 50594) trophozoites is indicated by the white arrow. The brown arrow shows the remnants of the aspirated *P. caudatus* prey. (**A**) Overlay of DIC and DAPI. (**B**) Overlay of DIC, DAPI and 40 nm beads. (**C**) Overlay of DIC, DAPI, 40 nm beads and CellMask actin red. (**D**) Overlay of DIC, DAPI and CellMask actin red, (**E**) DAPI, CellMAsk actin red and 40 nm beads. (**F**) Overlay of CellMask actin red and 40 nm beads. (**G**) Overlay of DAPI staining and 40 nm beads (green). (**H**) Overlay of DAPI staining and CellMask actin red.

In the pre-cyst of *Colpodella* sp. (ATCC 50594) the food vacuole contained the nucleus and kinetoplast of *P. caudatus* (Figure 6A) and the green 40 nm nanoparticles (Figure 6B,G). The CellMask actin distribution in the pre-cyst was observed in the cytoskeleton of the pre-cyst and in the remnants of the anterior region of the *Colpodella* sp. (ATCC 50594) trophozoite (Figure 6D–F,H). We previously reported that initial contact is made by *Colpodella* sp. (ATCC 50594) trophozoites using a myzocytic aperture located posterior to the pseudoconoid followed by uptake of the prey’s membrane, dissolution of the membrane and aspiration of the prey’s cytoplasmic contents [24]. The process of myzocytosis is a sequential process and not a direct piercing of the prey’s plasma membrane by the predator.

**Figure 7:**
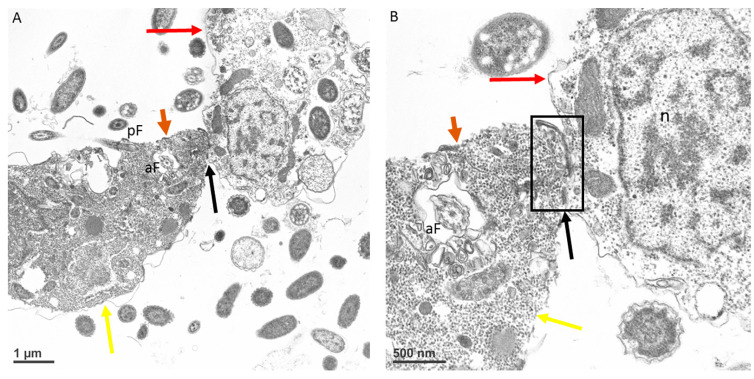
Transmission electron microscopy showing *Colpodella* sp. (ATCC 50594) (yellow arrow) and *P. caudatus* (red arrow) in myzocytosis. The point of attachment is posterior to the tip of the rostrum in the pseudoconoid (brown arrow) (**A**). The point of attachment (boxed area, black arrow) is shown in an enlargement of panel A, indicating the tight contact and microtubular organization at the contact point (**B**). Scale bars, **A** (1 µm), **B** (500 nm). aF, anterior flagellum, and n, nucleus.

**Figure 8:**
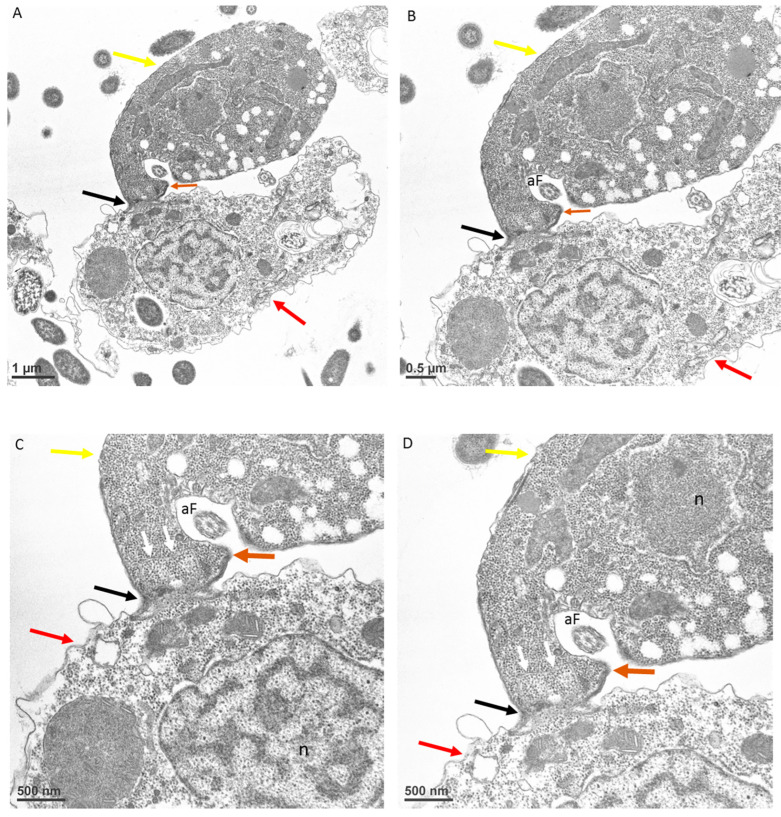
Transmission electron microscopy showing a young trophozoite of *Colpodella* sp. (ATCC 50594) (yellow arrow) and *P. caudatus* (red arrow) in myzocytosis. The point of attachment is posterior to the tip of the rostrum in the pseudoconoid (brown arrow) (**A**). The tight contact at the point of attachment (black arrow) showing that the membrane of *P. caudatus* is drawn into the cytoplasm at the apical region of *Colpodella* sp. (ATCC 50594) shown in an enlargement of panel A (**B**). The white arrows in panels **C** and **D** show the plasma membrane and cytoplasm of the prey pulled into the predator, indicating that the membrane of *P. caudatus* is not pierced during initial contact. Scale bars, **A** (1 µm), **B** (0.5 µm), **C** (500 nm) and **D** (500 nm). aF, anterior flagellum, and n, nucleus.

**Figure 9:**
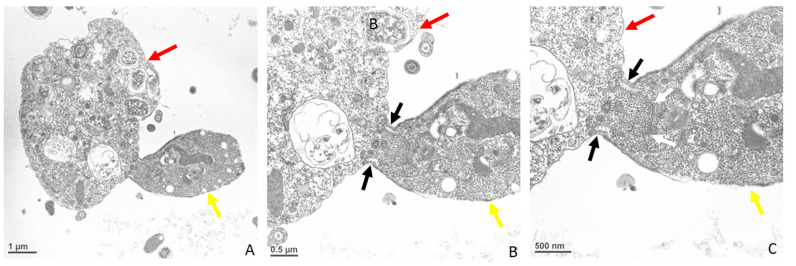
Transmission electron microscopy showing a young trophozoite of *Colpodella* sp. (ATCC 50594) (yellow arrow) and *P. caudatus* (red arrow) in myzocytosis. The thickened pellicle of *Colpodella* sp. (ATCC 50594) is shown surrounding the plasma membrane and cytoplasm of *P. caudatus* being pulled into the cytoplasm of the predator (**A**). Panel A is enlarged to show details of the attachment (black arrow) in panels **B** and **C**. The white arrows in panel C show the plasma membrane of the prey pulled into the predator, indicating that the membrane of *P. caudatus* is not pierced during initial contact. Scale bars, **A** (1 µm), **B** (0.5 µm), and **C** (500 nm).

At the point of contact between predator and prey, there is a reorganization of microtubules (Figure 7A,B) followed by uptake of the prey’s plasma cell membrane (Figure 8A–D, white arrows). The tip of the pseudoconoid containing the rostrum in the young trophozoite of *Colpodella* sp. (ATCC 50594) shown in the TEM micrograph is pointed away from the contact site (brown arrow), while the attachment site (black arrow) is in direct contact with the prey’s membrane. From this contact site, aspiration of the cytoplasmic contents of the prey occurs following dissolution of the prey’s plasma membrane. The prey’s (red arrows) membrane is pulled in further into the predator’s (yellow arrows) cytoplasm, as shown in Figure 9A and enlarged in 9B and 9C (white arrows). Extended membrane rims of *Colpodella* sp. (ATC C 50594) are seen surrounding the prey’s plasma membrane (Figure 9B,C, black arrows), forming the tubular tether.

## 4. Discussion

*Colpodella* species feed on their prey using myzocytosis. Juvenile *Colpodella* sp. (ATCC 50594) trophizoites have been observed feeding on mature *P. caudatus* trophozoites, and mature *Colpodella* sp. (ATCC 50594) trophozoites in culture feed on juvenile *P. caudatus* trophozoites. In culture, *Colpodella* sp. (ATCC 50594) trophozoites can be identified without prey attachments and trophozoites have been observed to detach from their prey after initial attachment and persist in culture. In this study, we investigated the hypotheses that unattached trophozoite stages would take up nutrients through means other than myzocytosis and that aspirated contents from the prey would be degraded before uptake and incorporation into the posterior food vacuole. We also wanted to identify the point of contact between predator and prey at the initial stages of myzocytosis. Fluorescent particles of 4 µm, 2 µm 100 nm and 40 nm were used to investigate the size of aspirated particles taken up during myzocytosis. Free, unattached, active young trophozoites of *Colpodella* sp. (ATCC 50594) and mature trophozoites were observed in culture. The size of aspirated nutrients has not been reported for *Colpodella* species, and in our previous light microscopy and ultrastructural studies, particulate nutrients were not observed in the pre-cysts and cysts of *Colpodella* sp. (ATCC 50594) [23,24,25].

In culture, young trophozoites of *Colpodella* sp. (ATCC 50594) can feed on different stages of *P. caudatus* trophozoites, forming tubular tethers that are used for aspirating cytoplasmic contents. In unattached *Colpodella* sp. (ATCC 50594) trophozoites, DAPI-stained contents were identified in the cytoplasm, suggesting that nutrient uptake can take place independently of myzocytosis. Results of nutrient uptake experiments showing uptake of 40 nm and 100 nm beads into unattached trophozoites of *Colpodella* sp. (ATCC 50594) confirmed uptake by endocytosis. Although not considered bacteriotrophic, bacteria from culture were observed in the cytoplasm of *Colpodella* sp. (ATCC 50594) trophozoites. However, fluorescent *E. coli* BioParticles were not taken up. Aspirated cytoplasmic contents of *P. caudatus* were observed in the tubular tether between predator and prey during myzocytosis. Intact kinetoplast and nuclei of *P. caudatus* were observed in the posterior food vacuole of *Colpodella* sp. (ATCC 50594) in the trophozoite and pre-cyst stages of the life cycle (Figure 5 and Figure 6). These data suggest that the kinetoplast and nuclei can be squeezed through the tubular tether during aspiration, further indicating that aspirated contents may not have to be digested before aspiration. Nuclei reportedly deform when cells squeeze through tight or constricted spaces [26]. Based on light and transmission electron microscopy, it appears the diameter of the tether may vary, just as the lengths of the tether vary [24], thus allowing nutrient content of varying sizes to be aspirated.

Among the Apicomplexa, trophozoites of blastogregarines and archigregarines attach to their host cells using the mucron, through which, nutrients are obtained by myzocytosis [16,17]. The apical complex organelles and the mucron are present throughout the life cycle. In pathogenic Apicomplexa, contents of apical complex organelles are secreted and participate in junction formation, entry into the host cell and parasitophorous vacuole (PV) formation in organisms that form the PV [6,27]. *Colpodella* species do not invade their prey, and so differ from *Selendium* species where the trophozoites obtain nutrients by myzocytosis from their host cells [17,27]. *Cryptosporidium* species interact with their host cells by forming a feeder organelle, composed of actin at the site of interaction and obtain nutrients in a manner similar to the archigregarines and *Colpodella* species [27]. The epimerite plasma membrane in gregarines forms a border with the invaginated host cell plasma membrane [16,17] similar to the tight interaction observed between *Colpodella* sp. (ATCC 50594) and the prey’s membrane in [24] and the current study. The sequential progression of myzocytosis involving attachment to the prey, the uptake of the prey’s membrane into the cytoplasm of the predator and dissolution of the membrane to permit aspiration of the prey’s cytoplasmic contents into the posterior food vacuole argue against phagocytosis and trogocytosis [24,25]. Moreover, the presence of a “myzocytotic apparatus” posterior to the tip of the pseudoconoid demonstrates that attachment is not followed by the piercing of the prey’s plasma membrane by the pseudoconoid. Rather, the uptake of the prey’s membrane followed by its dissolution to create a channel through which cytoplasmic contents are aspirated is in agreement with the myzocytotic process described for *Selenidium orientale* where the cytostome is used and can re-open for intermittent feeding [28]. It is unclear if the “myzocytic aperture” in *Colpodella* sp. (ATCC 50594) is within the opening of the pseudoconoid or a separate structure such as the cytostome or micropore. Myzocytosis has been described as a type of endocytosis [29] and as a form of “apical phagotrophy” [17]. This form of nutrient acquisition prevalent among free-living predatory alveolates, archigregarines and possibly *Cryptosporidium* species may be the mode of feeding used by the common ancestor of the Apicomplexans [17]. Myzocytosis for predation as described for *Colpodella* species and *V. pontica* [3] represents the ancestral form of feeding among the apicomplexans, with *Cryptosporidium* species serving as the link between predatory feeding, extracellular parasitism and intracellular parasitism following host cell invasion [28].

The IMC is described as a barrier to endocytosis in Apicomplexa, with the micropore serving the role of an endocytotic apparatus, and the cytostome consists of proteins that are associated with endocytosis [12]. The observation that *Colpodella* sp. (ATCC 50594) trophozoites perform endocytosis, with the uptake of 100 nm and 40 nm beads (Figure 1, Figure 2 and Figure 3), suggests that endocytosis may occur at the apical end where regions of the plasma membrane can attach with the membrane of the prey without obstruction by the IMC. This same area may be used for nutrient acquisition during myzocytosis. The size and shape of the 4 µm and 2 µm particles may have hindered uptake by *Colpodella* sp. (ATCC 50594) and *P. caudatus,* since the larger particles were not observed in the cytoplasm of the protists. The rhoptries and micronemes in *Colpodella* species are emptied during myzocyosis and may participate in the digestion of the prey’s membrane and the uptake and digestion of aspirated nutrients. The unexpected observation in this study of intact nuclei and kinetoplast of *P. caudatus* in the cytoplasm of free unattached trophozoites and in food vacuoles of *Colpodella* sp. (ATCC 50594) in pre-cyst stages demonstrates that undigested particulate, as well as digested soluble nutrients, can be taken up by *Colpodella* sp. (ATCC 50594) trophozoites in diprotist culture, contrary to our hypothesis. A major challenge to performing nutrient uptake studies in diprotist culture with nanoparticles is the presence in the culture of more *P. caudatus* than *Colpodella* sp. (ATCC 50594) and the presence of large amounts of bacteria. We are currently investigating synchronization protocols to enrich for each protist and eliminate bacteria. This will facilitate quantitating particle uptake at each stage of the life cycle for each protist in future experiments, as well as performing endocytosis inhibition studies. The synchronization of life cycle stages will also aid the identification of proteins involved in endocytosis. The cytoskeleton participates in tubular tether formation, as we showed previously that cytochalasin D treatment distorts the tubular tether [24]. CellMask actin red distributes over the tether (Figure 5), in agreement with actin involvement. Additional studies will be required to identify proteins associated with endocytosis and to map their distribution in free trophozoites, trophozoites engaged in myzocytosis and in the pre-cysts and cyst stages of the life cycle. Due to the phylogenetic closeness of *Colpodella* species to the pathogenic Apicomplexa [1,2] and the recent data showing the association of endocytosis with artemisinin resistance in *Plasmodium falciparum* [12,30], the events of nutrient acquisition seen in the free-living alveolate relatives of pathogens such as *Plasmodium* species may provide clarity for understanding nutrient acquisition and the role of the food vacuole during intracellular infection.

## 5. Conclusions

Our data show for the first time that *Colpodella* sp. (ATCC 50594) use myzocytosis and endocytosis for nutrient acquisition during growth in diprotist culture. Unattached *Colpodella* sp. (ATCC 50594) trophozoites take up nutrients in culture independently of myzocytosis and may attach and feed intermittently before detaching to seek other prey. The presence of a “myzocytotic aperture”, most likely a structure similar to a cytostome, may permit intermittent feeding. During myzocytosis, *Colpodella* sp. (ATCC 50594) trophozoites do not pierce the plasma membrane of its prey to obtain nutrients. Following attachment, the plasma membrane of the prey is taken up into the cytoplasm of *Colpodella* sp. (ATCC 50594), followed by dissolution of the membrane, to form a channel through which the cytoplasmic contents of the prey are aspirated into the posterior food vacuole. Nutrients as large as intact nuclei and kinetoplasts were identified in the posterior food vacuole of *Colpodella* sp. (ATCC 50594), suggesting that the organelles can be squeezed through the tubular tether with the cytoplasmic aspirate and deposited in the posterior food vacuole. Fluorescent nanoparticles of 40 nm and 100 nm were taken up by *Colpodella* sp. (ATCC 50594) and *P. caudatus*. Nanoparticles taken up by *P. caudatus* were aspirated by *Colpodella* sp. (ATCC 50594) during myzocytosis. Fluorescent *E. coli* BioParticles were only taken up by *P. caudatus*. In future studies, endocytosis inhibition will be performed to gain insights regarding the cytoskeletal components involved in nutrient uptake and proteins involved in endocytosis will be identified. The use of synchronized life cycle stages of predator and prey in future nutrient uptake studies will permit the quantitation of beads taken up by both protists. Myzocytosis for predation may be the ancestral mode of nutrient acquisition among the Apicomplexa.
